# Co-Designing a National Family Handbook for Childhood Brain Tumor

**DOI:** 10.3390/children12091126

**Published:** 2025-08-26

**Authors:** Melanie L. Rolfe, Evonne Miller, Liesje Donkin, Stuart Ekberg, Natalie K. Bradford

**Affiliations:** 1 Cancer and Palliative Care Outcomes Centre at Centre for Children’s Health Research, School of Nursing, Queensland University of Technology, 62 Graham Street, South Brisbane, Brisbane, QLD 4101, Australia; natalie.bradford@qut.edu.au; 2 Design Lab, School of Design, Queensland University of Technology, Z4, Kelvin Grove Campus, Creative Industries Precinct, Musk Avenue, Kelvin Grove, Brisbane, QLD 4059, Australia; e.miller@qut.edu.au; 3 Department of Psychology and Neuroscience, School of Clinical Sciences, Auckland University of Technology; 90 Akoranga Drive, Northcote, Auckland 0627, New Zealand; liesje.donkin@aut.ac.nz; 4 Caring Futures Institute, College of Nursing and Health Sciences, Flinders University, Sturt Road, Bedford Park, Adelaide, SA 5042, Australia; stuart.ekberg@flinders.edu.au; 5 Viertel Cancer Research Centre at Cancer Council Queensland, 553 Gregory Terrace, Spring Hill, Brisbane, QLD 4006, Australia

**Keywords:** community-based participatory research, co-design, psychosocial functioning, central nervous system neoplasms

## Abstract

Background/Objectives: Parents report unmet information needs relating to childhood brain tumors. Existing research shows that providing information to families supports self-efficacy and well-being. The project therefore aimed to co-design resources tailored to the informational needs of families navigating childhood brain tumors in Australia. Methods: Mixed methods were used across multiple phases. A landscape analysis in Phase 1 confirmed the gap in Australian resources as well as the identification of international resources suitable to inform local solutions. Following the Double Diamond Design Framework, subsequent phases of the project aimed to discover and define the problems faced by families before developing and delivering the solution. Parents of children with brain tumors participated in a journey mapping workshop, content adaptation through feedback, and an online survey to determine the preferred delivery mode of information. Clinicians provided iterative feedback as the resource was developed and refined. Results: Nine mothers participated in journey mapping and iterative adaptation of the resource along with twelve clinicians. There were 46 respondents to the survey, which identified a preference for multi-modal delivery of information, and 23 clinical and consumer reviewers in the final revision phase. The process of adaptation is presented, providing transparency on the development of this national resource. Conclusions: The use of self-efficacy theory and co-design was pivotal in this project. Integration of concepts from self-efficacy moves beyond simply presenting information to empowering the audience to feel capable of the task ahead of them. Co-design ensured the content and tone of the resulting resource are fit-for-purpose from the perspective of both clinicians and consumers. The resource is available as a physical book, digital resource, and audiobook and disseminated through children’s hospitals, professional networks, and brain tumor support groups.

## 1. Introduction

When a child is diagnosed with a brain tumor, it impacts every facet of the family’s life. Families must frequently reorganize daily life to accommodate treatment, with long hospital admissions often requiring separation of family members, changes to parental work schedules, and disruption to education and regular activities for the child and their siblings [1]. The diagnosis of a brain tumor brings exposure to potentially traumatic medical experiences and emotional turmoil related to threats to life and well-being [2]. Because of their location, brain tumors and their treatment can cause ongoing disability such as seizures, personality changes, and physical impairments that can impact independence with activities of daily living such as eating and mobility [3,4]. Children with brain tumors frequently also experience psychological impacts, and these can persist into adulthood [5]. For parents, the ongoing nature of these potential impacts means that diagnosis is accompanied by concerns for managing the difficulties of treatment, along with concerns for the child’s survival and quality of life after treatment. These concerns negatively impact the functioning of the family unit, sibling and parental wellbeing, and the family’s wider support network [6,7,8,9,10].

Despite recognition of the psychological impact of serious childhood illnesses, such as a brain tumor diagnosis, families and clinicians frequently report unmet needs for support and information. In a 2023 survey of clinicians, over 70% believed families accessing the pediatric medical system should receive information on preparing for potentially traumatic medical experiences; however, less than 25% reported offering this [11]. Parents frequently report an unmet need for mental health support for their own well-being [12,13,14,15] and request education on navigating their own and their child’s mental health related to the medical experience [16]. Parents frequently report they are not offered any psychological support for themselves [12], and only 24% of clinicians indicate they routinely give parents with seriously ill children information on recognizing signs of heightened psychological needs [11]. This pattern of inadequate recognition of the psychological impact and needs of parents is also found in the context of Australian families navigating childhood brain tumors [1,17].

The interplay of parent, child, and family functioning in the context of childhood brain tumors is complex. Parents and children have independent contributions to distress, and each member of a parent-child dyad impacts the other’s mental health [18,19]. As well as the inevitable distress associated with their child’s health, parents report modifiable factors of their experience that impact their level of stress. These include communication with health professionals, access to understandable information on the tumor and treatment, preparation to support their child through medical procedures and to support siblings, and understanding when and how to access mental health support for themselves and their children [20,21]. Provision of such information at the right time may support positive health behaviors, facilitate communication with care teams, better prepare children for medical procedures, and navigate social and psychological components of family life. Improving these modifiable components of distress associated with a childhood brain tumor may positively impact quality of life for the child and parent.

Self-efficacy is an important concept in health education, enabling improved uptake of positive health behaviors and bridging the gap between provision of knowledge and action [22]. Self-efficacy theory, first developed by Bandura in the 1960s, outlines cognitive and behavioural components that contribute to a person’s likelihood of initiating and maintaining behaviors [23]. Specifically, self-efficacy theory proposes that a person’s expectation that they are capable of trying and succeeding in a behaviour informs whether they attempt that behaviour [23]. Developing this self-belief, therefore, is a route to behaviour change [23].

Self-efficacy can be developed via experiences of mastery, vicarious experiences of success through social models, social persuasion, and down-regulating stress reactions to the behaviour [24]. Bandura (1994) also highlights the importance of persistence and normalization of struggle in promoting self-efficacy: ‘Insulation from problematic situations leaves one ill-prepared to cope with potential difficulties.’ (p76, [24]). Bandura’s more recent revisions of the theory identify that in addition to a person’s belief in their own ability (self-efficacy belief) and belief in a positive outcome (outcome expectancies), goals, facilitators, and impediments are also contributing factors to behaviour change [23].

In pediatric cancer research, self-efficacy is a demonstrated mediating factor between family-centered care and psychological well-being; when parents receive family-centered care, self-efficacy is heightened, resulting in positive psychological outcomes [25,26]. Salvador (2019) has demonstrated that appropriate information provision can support this self-efficacy and psychological well-being [26]. The provision of information, therefore, is one way that medical systems can enable the self-efficacy and psychological well-being of families. Where the content aligns, information provision normalizes struggle and enables social modeling and persuasion [24]. As our earlier research identified a gap in such informational resources [1], this project aimed to co-design such a resource with clinicians and families with lived experience supporting a child diagnosed with a brain tumor.

## 2. Materials and Methods

Utilizing co-design methodology, the project embedded end users—parents of children with brain tumor—in the development of the ultimate resource [27,28]. Co-design, the process of designing a resource with the end users, results in better-suited resources with increased uptake by end users [28]. The co-design methodology followed the Double Diamond framework, categorically building through the phases of discovering and defining the problem before developing and delivering the solution [29]. Quantitative and qualitative methods were used across five phases to achieve the project aims (see Table 1). Ethics was approved through the Children’s Health Queensland Human Research Ethics Committee (HREC/19/QCHQ/53816).

### 2.1. Phase 1: Landscape Analysis of Health Information

#### 2.1.1. Objective

To identify resources available to families of children with brain tumors in Australia.

#### 2.1.2. Methods

An online search was undertaken to identify family-facing resources relevant to childhood brain tumor in Australia. Consultation occurred with clinicians working in childhood brain tumor clinical and community services to confirm findings of online searches. Identified resources were collated in electronic and hard copy and reviewed for suitability.

### 2.2. Phase 2: Co-Design

#### 2.2.1. Objectives

1. Understand un-met needs of families navigating childhood brain tumor
2. Identify key aspects for solutions to identified needs
3. Develop content for solutions

#### 2.2.2. Participants

Participants were parents of children treated for brain tumors (consumers) who had participated in previous research at a major children’s tertiary hospital [21]. A member of the original study team, known to the participants, contacted each participant via email and provided written information about the study. After having time to read the information and consider their participation, participants emailed the current study team to give their consent.

#### 2.2.3. Methods

In consideration of the sensitive nature of the topic, participants were able to elect to participate in one or both activities of a group online workshop or a written workbook. The Needs and Aspirations for Design and Innovation model (NADI model [30,31,32]) informed the structure of the workshop and written workbook elements. Connecting to self-efficacy theory’s inclusion of goals and needs as contributors to actioning new health behaviour [22,23], the NADI model identifies four deepening levels of understanding an issue and solution for effective design: solutions, scenarios, goals, and themes. Journey mapping, which involves identifying important milestones relating to an experience across a timeline [33], was utilized to understand the deepest levels of themes and goals; participants were asked to describe the issues they faced across timepoints in their experience (‘themes’ in the parlance of NADI) and what support they needed at these times (‘goals’; reported elsewhere) [20]. Participants were then asked to identify possible solutions. Finally, resources identified in Phase 1 (Landscape Analysis) were presented for exploration as a starting point to design a resource suitable for use in Australia. The online workshop was audio-recorded for analysis.

#### 2.2.4. Analysis

The transcription and recording of the workshop were analysed using a narrative approach [34], with verbatim quotes used in the presentation of findings.

### 2.3. Phase 3: Identification of Delivery Preferences

#### 2.3.1. Objective

To identify national preferences for the mode of information and resource delivery.

#### 2.3.2. Participants

National charities that support families of children with brain tumors advertised their email database and via social media.

#### 2.3.3. Methods

An online survey was developed in secure online software, Qualtrics XM 2024. The survey asked for demographic and clinical information, such as stage of brain tumor experience, brain tumor type, and in which state treatment was received. Participants were asked to give a score from 0 to 10 to indicate the usefulness of various formats of information delivery and could respond to an open-text question with any other comments relating to psychosocial service need and preference. Other questions in the survey related to preferences for a service model and are reported elsewhere.

#### 2.3.4. Analysis

Descriptive statistics were used to analyse quantitative survey data. Proportions and means were calculated. Analysis was informed by the principles of content analysis [35], synthesizing the manifest data.

### 2.4. Phase 4: Review of Resource

#### 2.4.1. Objective

To refine the content, language, and design of the identified resource in line with end-user preferences.

#### 2.4.2. Participants

Consumers and clinicians involved in earlier phases were invited to continue their involvement in this phase. Local clinicians identified colleagues in other states who perform similar roles, and the resource was promoted at a national childhood cancer conference to recruit additional reviewers.

#### 2.4.3. Methods

Consumers and clinicians involved in earlier phases were invited to review the adaptation of a resource identified in Phase 1 (Landscape Analysis) for appropriateness and adequacy of content, suitability of language, and design elements. The resource was distributed via email for feedback, which was iteratively integrated. Findings of each previous phase iteratively informed the resource as the phases progressed.

Core adaptations that informed the final resource are reported in an amended version of the Framework for Reporting Adaptations and Modifications to Evidence-based interventions (FRAME) [36]. See Supplementary Table S1 for modifications to FRAME (Supp1). The resource was professionally graphically designed and circulated again to stakeholders for comment. The second round of review extended beyond the initial participants to broader professional stakeholders across Australia.

### 2.5. Phase 5: Dissemination

#### 2.5.1. Objectives

To identify a sustainable mode of dissemination for the developed resource.

#### 2.5.2. Methods

Utilizing established professional networks, a national charity partner was approached to support dissemination. After initial informal communication, a proposal was submitted to the board for their consideration to host the digital version on their website. National participants from Phase 4 were asked for their interest in distributing the resource to families they support.

## 3. Results

### 3.1. Phase 1: Landscape Analysis of Health Information

The online search found no existing Australian resources for family-facing education and information on childhood brain tumor. This finding was confirmed through consultation with clinicians and community support groups. One international resource was identified—the Pediatric Brain Tumor Handbook, published by the Brain Tumor Foundation of Canada [37]. The foundation gave their permission for this resource to be used for an Australian adaptation using entirely new language.

### 3.2. Phase 2: Co-Design Workshop

Nine participants (all mothers) consented to participate in the co-design process. Six of these participants took part in an online workshop, as well as contributing written data through the workbook. Three mothers contributed written data only in the workbook (Table 2).

The journey mapping facilitated through the online workshop and workbook elicited various needs across the trajectory of brain tumor treatment (reported in full elsewhere) [20]. Participants highlighted the need for universal information delivery, independently of researchers, who subsequently shared the chapter titles from the Canadian resource for comment. The need for an Australian version was strongly endorsed by participants.

The chapter titles of the Canadian resource were discussed and amended to address needs identified through journey mapping. Notable edits included changing the title of the ‘Palliative Care’ chapter to ‘Quality of Life Care’, as one participant stated, ‘It might be helpful to not have ‘palliative care’ in the title…I skipped over that […] because I see the word ‘palliative’ […] I would never read that chapter.’ (P3). Another agreed, ‘I read it as ‘if your child’s terminal’ (P4). These comments highlighted a contrast between the intent of the chapter, to encourage people early in the trajectory to consider referral to the palliative care team if their child’s condition could be considered ‘life-threatening’ and the reality of a parent avoiding the content due to the language used in the chapter title holding different meaning to them.

After the online workshop, one researcher (MR) edited the resource in line with workshop outcomes. Consumer edits included acknowledging that this experience is ‘scary’ (P5), making the language a ‘bit more real’ (P5) with a ‘peer-to-peer tone that acknowledges the seriousness’ (P3), and additional information on ‘where to go [online] for your first info[rmation] dump’ (P3), and asking for help and setting boundaries with social support networks. In addition, a chapter was included on family relationships, as well as a letter template to send to the child’s school. Participants voiced information needs specific to their child’s situation relating to fertility impacts and real-world impacts of disability, such as the ability to drive with vision loss. In response, fertility was highlighted as an area for clinical conversation early in the resource when making treatment decisions, and spaces were incorporated to write questions, notes, and specific information about the child’s diagnosis and treatment in the resource.

In the workshop, participants and researchers also discussed the scope of the resource, and the consensus was reached that where there was not capacity to go into detail on a topic, there should be links to reputable online sources, for example, details of specific tumor types. Components that were deemed out of scope for this project were also raised, such as opportunities for learning from the lived experience narratives of others:

*For me, I was really looking for those sorts of stories—like real stories, rather than just reading about different tumors online. I was looking for stories that weren’t necessarily positive but just other people’s experience of the type of treatment we were about to go through. (P5)*

Participants suggested podcasts would be one mode of making these stories accessible to a wide audience. While this component is not within the scope of this project, lived experience was embedded in the handbook through the use of participant quotes and reference to the group who co-designed the handbook.

The workshop resulted in consensus on the chapter topics and the peer-to-peer tone of the handbook. To address the issue of depleted time and mental energy during treatment, participants recommended that the resource be available in multiple formats, including a physical book, audiobook, and electronic formats.

### 3.3. Phase 3: Identification of Resource Delivery Preferences

The online survey was completed by 46 parents of children with brain tumors: 39 mothers and seven fathers. Diagnoses occurred from 2007 to 2024 (year of survey). Most participants were bereaved (n = 14, 30%) or attending outpatient appointments for follow-up with their child (n = 13, 28%), and the sample was representative of the Australian population in terms of regionality (see Table 3). Of the 46 survey participants, 37 participants reported their preferences for information delivery format, reported in Table 4.

Twenty-one participants also reported preferences in the free-text comment box. Responses relating to information delivery included a desire for easily accessible online resources such as podcasts or videos for when ‘parents don’t feel like talking or interacting with medical professionals’ (SP2). Most comments related to desired topics of information, such as long-term and late effects, available emotional support services, and how to access these. At all stages, families wanted strategies and resources to support ‘navigating life with a child diagnosed with a brain tumor’ (SP1) and managing the ‘dual responsibility’ (SP1) of hospital treatment with one child while there are ‘other children at home who needed our attention, love, and support’ (SP1). Bereaved families wanted more guidance on managing day-to-day life and the psychological impact of losing a child (SP1) and information they did not ‘have to go looking for’ (SP20). Parents also indicated an unmet need for narrative-based education, or ‘learning through the real-life journeys of others’ (SP9). One suggestion for this was the development and sharing of a ‘typical path’ (SP8) through combining people’s stories with childhood brain tumor.

Following the results of this phase, the handbook has been recorded as an audiobook available through mainstream podcast hosting services, and an electronic version is hosted online. Although development of an interview-style podcast is out of scope for this project, findings highlight there is a desire and need for this.

### 3.4. Phase 4: Iterative Review of Resource

Consumer and clinical participants from earlier phases of the study were invited to participate in the review of the resource. Six consumer participants, eight clinical participants, and the research team contributed to the first round of revisions, which were staggered and iterative. Some participants and researchers reviewed the resource multiple times.

MR and NB presented major findings from the workshop to the clinical co-designers. They were then given an electronic copy of the draft handbook to review and edit, with the option of editing the full document or chapters within their specific clinical area. This process resulted in changes to language describing clinical interventions and the addition of sections relating to the National Disability Insurance Scheme (NDIS), feeding, and exercise.

The consumer co-designers were given an electronic copy of the draft handbook to review. They contributed their feedback individually using track changes and comments on the Microsoft Word document. As a result, the language was again simplified wherever possible, and where complex language could not be avoided, it was explained in text. The consumer group also contributed poems and quotes that they wished to share with other families navigating their child’s brain tumor diagnosis and treatment.

Between clinical, research, and consumer revisions, there were a total of 12 iterative versions before being finalized and shared with the publisher. Clinical reviewers to this point are acknowledged as co-authors in the handbook, and consumer reviewers to this point who wished to be acknowledged are named in the ‘acknowledgments’ section of the handbook.

Once the publisher had formatted the book for publication, a printed copy was circulated to a national charity for childhood brain tumor and clinicians across Australia for final review to ensure the content was inclusive of any regional variations. Table 5 describes the reviewers for this phase. Table 6 describes the nature of major changes to the handbook across phases.

### 3.5. Phase 5: Dissemination

A national childhood brain tumor foundation agreed to host the digital resources online, and these can be found at www.brainchild.org.au (accessed on 12 August 2025). The audiobook version is available on podcast hosting services including Spotify (https://open.spotify.com/show/7iUoaQd7RYrCIQS4svVVuj?si=f66a4eca6f214fc0, accessed on 12 August 2025) and Apple (https://podcasts.apple.com/us/podcast/the-australian-family-handbook-for-childhood-brain-tumour/id1832404208, accessed on 12 August 2025), under the title ‘The Australian Family Handbook for Childhood Brain Tumour’. Physical copies were distributed to Clinical Nurse Consultants in each state who support children with brain tumors and their families, with the instruction to offer these to every family of a child with a benign or malignant brain tumor. The handbook includes a link for families to give feedback on the resource, which will inform subsequent editions. In this way, the co-design process will continue as other needs are identified through feedback from a broad range of stakeholders.

## 4. Discussion

This co-design project iteratively developed a family-facing resource for childhood brain tumor through identifying a gap (Phase 1), identifying and designing a desired solution with end users (Phases 2–4), and embedding its distribution in the Australian health system (Phase 5). The project outcome has addressed consumers’ calls for ‘peer-to-peer’ toned communication, which supports their understanding of brain tumors, treatment, long-term treatment- and tumor-related effects, and the psychological well-being of all members of the family. As parents quickly become experts in their child’s condition, this project has highlighted and filled the need for standardized, accessible resources that equip parents with clinically complex information as it is needed.

Our earlier research identified parents who desired more information to empower them to help themselves and their families [1,21]. ‘Self-help’ health education and information resources are effective in supporting health behaviour change when self-efficacy is targeted alongside the provision of health information [38]. Alongside factual information on these topics, the handbook embeds information that supports the two components of self-efficacy: belief in one’s own ability to achieve the task (self-efficacy belief) and belief that one’s actions can influence an outcome positively (outcome expectancy) [23]. These elements of self-efficacy are targeted through connection to the lived experience of others, normalizing struggle and sharing stories of success throughout the book. These elements build self-efficacy through social modelling; seeing people similar to us navigate and succeed at experiences [39]. Lived experiences are embedded through reference to the group who co-designed the book, poems and quotes that this group proposed for the resource, and direct quotes from the group. Considering these aspects, the handbook can be viewed as a ‘self-help’ resource, comprised of information relating to family relationships, mental health, and supporting preparation for medical procedures.

When workshop participants were asked what was needed for their own psychological well-being, parents frequently reported components relating to their child’s health. These responses highlighted that being able to support their child’s medical and psychological well-being is central to their own well-being. Information in the handbook relating to brain tumors and their treatment therefore support parents’ psychological well-being through targeting their self-efficacy in supporting their child. This information supports quantitative findings of reciprocal relationships between parent and child well-being in pediatric brain tumor [40].

More broadly, this work contributes to the literature on co-design practices in pediatric healthcare. While co-design in health care is becoming more commonplace, the processes for this are not always well described [41]. Here we have outlined not only the multiple phases where consumers’ needs and solutions were shared, but also how the process of co-design iteratively shaped the resource. Recognizing that consumers are partners in healthcare, this project engaged consumers from ideation through to completion, and this contributed to a resource that is digestible and suited to this audience. The use of the double-diamond framework [29] served to first deepen understanding of nuanced factors of the information gap before designing solutions and highlighted the phase of ‘distribution’ as a core component of the design process.

The embedding of both consumer and professional perspectives across phases is a core strength of this work, enabling an end product that is endorsed by professionals and recommended by consumers. A limitation of our consumer sample is that it is small and pre-dominantly female, common in childhood cancer research [14]. Although women also make up the majority of care providers in childhood cancer [14], the resource aims to support all genders and to make information accessible to and supportive of family units working together through this adversity. As gender differences exist in parents’ psychosocial adjustment to childhood cancer [42], the suitability of the resource for different genders should be investigated in subsequent work to assess the uptake and effectiveness of the resource. Future editions should also investigate the need for content directed at siblings as part of the resource. The project aimed to mitigate the impacts of a small and potentially biased sample of consumers in the initial co-design phase by circulating the resource for review nationally with clinicians who work closely with a diverse range of families and tumor types.

In this project, consumers called for narrative education resources outside the scope of the current project. For instance, in Phase 2, families reported ‘looking for stories’ of others’ experiences to understand what the future might look like for their family. In Phase 3, parents ranked an interview-style podcast as their second-most preferred information delivery mode. Future projects should utilize this research to evidence the sharing of lived experience stories as an educational need for these families. Such educational materials can be considered health information resources that target self-efficacy through social modelling [39].

While the family handbook delivers information suitable for all families of children with brain tumor in Australia, participants also called for communication supports to facilitate clinical information exchange tailored to them. The group wanted tailored information regarding impacts of their child’s tumor and treatment on fertility, ongoing disability related to an acquired brain injury, and real-world impacts of their child’s disability, such as the ability to drive with vision loss. These topics were deemed most suitable for a separate resource such as a question [43,44] or discussion prompt list [45]. These kinds of resources suggest topics parents may want to bring up with their healthcare team and should be the target of future development in this field.

Overall, ‘the Australian Family Handbook for Childhood Brain Tumour’ has been thoughtfully co-constructed, taking an internationally recognised resource and tailoring it for the Australian audience through investigation of underlying needs and collaborative revision. The consideration of self-efficacy theory in its construction means that the end product does not just consider the information to be delivered but also ways to empower families with the belief that they are capable of the task that lies ahead of them. Embedding the resource in distribution channels across Australia ensures accessible information for all families of children with brain tumors. Embedding consumer insights across the resource acts as a reminder to families that others have walked this path before them, aiming to help them feel less alone as they find their way forward.

## Figures and Tables

**Table 1:** Phases of the Project Aligned to the Double Diamond Framework.

Phase	Methods	Double Diamond Element
1. Landscape Analysis of Health Information	Review of existing resources. In-person and email consultation through professional networks with health professionals	Discover
2. Co-Design	Workshop with parents, qualitative analysis of transcripts	Discover, define, develop
3. Delivery Preference Identification	Online survey with parents. Quantitative analysis	Develop
4. Resource adaptation and development	Drafting and critical review of resources. Stakeholder in-person consultation, analysis of field notes	Develop
5. Dissemination	Collaboration with key stakeholders and distribution channels	Deliver

**Table 2:** Phase 2 Participant Characteristics.

Characteristic	n (%) N = 9
Sex, female	9 (100%)
Remoteness of residence	
Metropolitan	4 (44%)
Capital City	1 (11%)
Non-capital City	3 (33%)
Regional	4 (44%)
Rural	1 (11%)
Stage of Treatment	
Finished acute treatment	8 (89%)
Bereaved	1 (11%)
Tumor Type	
Atypical Teratoid/Rhabdoid Tumor (ATRT)	1 (11%)
Glioma (High grade)	1 (11%)
Juvenile Pilocytic Astrocytoma (JPA)	2 (22%)
Medulloblastoma	2 (22%)
Retinoblastoma	1 (11%)
Not reported	2 (22%)

**Table 3:** Phase 3 Participant Characteristics.

Characteristic	n (%) N = 46
Sex, female	39 (85%)
Ethnic Identity	
Caucasian	38 (83%)
Caucasian/Aboriginal	2 (4%)
Asian	3 (6%)
Other	3 (6%)
Remoteness of residence	
Metropolitan	
Capital City	17 (37%)
Non-capital City	10 (22%)
Regional/Rural	17 (37%)
Remote	2 (4%)
State of Treatment	
Australian Capital Territory	1 (2%)
New South Wales	9 (20%)
Queensland	20 (43%)
Victoria	13 (28%)
Western Australia	3 (6%)
Stage of Treatment	
Newly diagnosed, awaiting treatment start	1 (2%)
On treatment, inpatient	1 (2%)
Outpatient appointments	13 (28%)
Finished acute treatment	
<12 months	2 (4%)
1–5 years	6 (13%)
>5 years	5 (11%)
Receiving palliative care	1 (2%)
Observation with no treatment	2 (4%)
Bereaved	14 (30%)
Tumor Type	
Benign	4 (9%)
ATRT	1 (2%)
Diffuse Intrinsic Pontine Glioma (DIPG)	7 (15%)
Ependymoma	2 (4%)
Glioblastoma multiforme	1 (2%)
JPA	10 (22%)
Medulloblastoma	12 (26%)
Other	7 (15%)

**Table 4:** Preferences for Information Delivery Mode.

Information Delivery Format (n)	Mean Score out of 10 (SD)
Website and/or online resource (36)	7.8 (2.3)
Interview Podcast (35)	7.5 (2.7)
Book (37)	7.1 (2.6)
Book and Audiobook (36)	6.9 (2.5)
Audiobook only (34)	5.1 (2.8)

**Table 5:** Phase 5 Participant Characteristics.

Characteristic	n (N = 23)
Clinician Type	
Clinical Nurse Consultant (pediatric oncology)	12
Allied Health (pediatric oncology)	4
Oncologist (pediatric)	2
Researcher/Advocate (pediatric brain tumor)	2
Consumer (pediatric oncology)	3
State	
Queensland	6
Victoria	4
South Australia	4
New South Wales	3
Western Australia	1
Tasmania	1
National	3

**Table 6:** Summary of Adaptations to the Resource in the Modified FRAME Framework.

What Need Informed the Change	Who Expressed the Need	Nature of Change	Who Decided	When	Level of Change	Goal of Change
Information on family relationships and parenting is needed	Parents	Added a chapter on this; References made to lived experience of this component.	Researchers	Workshop	Information	Self-efficacy belief; Outcome expectancy
Information on supporting their child through potentially traumatic medical procedures is needed	Parents	Added chapter on this; References made to lived experience of this component; Parents coached on calming themselves through the process	Researchers	Workshop	Information	Self-efficacy belief; Outcome expectancy; Downregulation
Parents report not being given all options relating to treatment decisions	Parents	Encouragement for parents to create a list of questions ahead of appointments with their healthcare team; Suggestions for questions embedded in the resource; Parents positioned as authorities in their child’s life and valuable members of their child’s healthcare team throughout the book	Researchers	Workshop	Information	Self-efficacy belief
Navigating appropriate support from informal support network identified as a difficult balance	Parents	Included a quote from a parent on navigating this component; Information given on strategies and resources for coordinating this support.	Researchers	Workshop	Information	Self-efficacy belief
Information on community services was delivered inconsistently; families frequently heard about these from other families	Parents	Included information on community services; References made to lived experience of this component.	Parents	Workshop	Information	Self-efficacy belief
Quality of support from schools is inconsistent	Parents	Included a letter to school as an appendix	Researchers	Workshop	Additional Resource	Pragmatic
Period after active treatment has ended is often a time of high psychological need for parents	Parents	Included information on this period; Encouraged early engagement with emotional and psychological support throughout the book. References made to lived experience of this component.	Researchers	Workshop	Information	Self-efficacy belief
Families unprepared for ongoing nature of brain tumor sequalae and late effects	Parents	Included information on this; References made to lived experience of this component.	Researchers	Workshop	Information	Self-efficacy belief
Term ‘Palliative care’ in chapter title is confronting and would deter people reading information that may be relevant	Parents	Re-named to ‘Quality of Life Care’, with introduction and definition of term ‘palliative care’ in first sentence	Parents	Workshop	Presentation	Pragmatic
Parents report symptoms of post-traumatic stress	Parents	Information on noticing traumatization and trauma-focused therapy included	Researchers	Workshop	Information	Self-efficacy belief
Some clinical language may be alarming for families	Clinician	Soften language (e.g., changed ‘protect from burns’ to ‘protect their skin’)	Clinician	Iterative Review	Presentation	Pragmatic
Centralized information would be useful	Parents	Resources developed	Researchers	Workshop, Online Survey	Delivery	Pragmatic; Self-efficacy belief components
Difficult to engage with long reading due to the demands of caring for their child	Parents	Offered audio version; Checked on preference for audio version	Researchers	Workshop; Online Survey	Delivery	Pragmatic

## Data Availability

No data are publicly available due to the identifiability of qualitative data.

## Supplementary Materials

The following supporting information can be downloaded at: https://www.mdpi.com/article/10.3390/children12091126/s1, Table S1: Adaptations to the FRAME Framework.
