# Supplementary material for: Co-Designing a National Family Handbook for Childhood Brain Tumor
Source: Children (Basel). 2025 Aug 26;12(9):1126. doi: 10.3390/children12091126 (PMC12468324; doi:10.3390/children12091126)
Supplement: Supplementary file 1 [file children-12-01126-s001.zip › children-3768310-supplementary.pdf]

Supplementary Table S1. Amendments to the FRAME Reporting Framework for Co-design

The Framework for Reporting Adaptations and Modifications to Evidence-based interventions (FRAME) [36] is designed to capture clinical adaptations to *interventions*, capturing for each amendment what was changed, when, why, and by whom [36]. As this project co-designed a resource, rather than made clinical changes to an intervention, these categories were amended to suit this purpose. The rationale for these amendments is outlined below.

FRAME Domain	Amended Domain	Rationale
What is modified	What is modified	No change
The reasons for the modification	What need informed the change	The separation of this question into the elements of underlying need and who identified this need reflects co-design practices of making amendments both because they have been specifically requested by end-users (reported at question 4), and because end-users have identified a need which researchers implement a solution to (reported in questions 1 and 2)
	Who expressed the need	
Who determined that the modification should be made	Who decided on the change	No change to content—language simplified
When and how in the implementation process the modification was made,	When in the co-design process the modification was decided	Language changed to reflect the co-design process
Whether the modification was planned/proactive or unplanned/reactive,	-	This question was omitted, as all modifications were in response to information acquired through co-design
At what level of delivery the modification is made	-	Not applicable as original question relates to delivery and receipt of the intervention
Type or nature of context or content-level modifications	Level of modification (information, presentation of information, delivery mode)	This question has kept the essence of identifying the context of the change but tailored the options to the co-design process
The extent to which the modification is fidelity-consistent	-	Not applicable as original question relates to clinical application of an intervention
-	Goal of Change	Added to reflect whether the change was pragmatic (in response to a specific issue raised with accessibility or information) or in aid of self-efficacy (verbal persuasion or vicarious experiences)
